# Mesenchymal stem cell-derived extracellular vesicles in the treatment of type 2 diabetes and its complications: current progress and future directions

**DOI:** 10.1186/s13287-026-04991-w

**Published:** 2026-03-29

**Authors:** Sha Zhang, Zong-Yu Zhang, Ruo-Nan Tang, Kai Zhang, Yu Fu, Hua Tian, Jing Ma, Yan Jin, Chen-Xi Zheng, Bing-Dong Sui

**Affiliations:** 1 College of Basic Medicine, Shaanxi Key Laboratory of Research on TCM Physical Constitution and Diseases Prevention and Treatment, Shaanxi University of Chinese Medicine, Xianyang, 712046 Shaanxi China; 2 State Key Laboratory of Oral & Maxillofacial Reconstruction and Regeneration, National Clinical Research Center for Oral Diseases, Shaanxi International Joint Research Center for Oral Diseases, Center for Tissue Engineering, The Fourth Military Medical University, Xi’an, 710032 Shaanxi China; 3 Department of Traditional Chinese Medicine, The First Affiliated Hospital of Fourth Military Medical University, Xi’an, 710032 Shaanxi China; 4 School and Hospital of Stomatology, Liaoning Provincial Key Laboratory of Oral Diseases, China Medical University, Shenyang, 110001 Liaoning China; 5 Department of Diabetes, School of Translational Medicine, Monash University, Melbourne, VIC 3004 Australia

**Keywords:** Type 2 diabetes, Diabetic complications, Mesenchymal stem cells, Extracellular vesicles, Bioactive materials, Artificial intelligence

## Abstract

Type 2 diabetes (T2D) and its complications represent a complex disorder involving multiple pathophysiological processes. Although conventional therapeutic approaches partially regulate blood glucose, they fail to fundamentally reverse disease progression or effectively prevent complications. This review summarizes the current research advance and challenges of using different forms of mesenchymal stem cell-derived extracellular vesicles (MSC-EVs) in treating T2D and complications. It begins with an introduction to the characteristics of MSC-EVs. Subsequently, the mechanisms and therapeutic prospects of natural MSC-EVs are analyzed, with a focus on their roles in inflammatory modulation, tissue regeneration, and improving insulin resistance. Engineering MSC-EVs, covering strategies including optimizing MSC culture conditions, modifying EV contents, and establishing MSC-EV delivery systems based on bioactive materials are then discussed, which boost EV yield and quality while enhancing therapeutic efficacy. Current challenges, including the limited yield and high heterogeneity of natural MSC-EVs, as well as issues related to long-term safety, immunocompatibility, and large-scale production of engineered MSC-EVs are finally overviewed, with emphasizing artificial intelligence in guiding future research directions. These summaries are crucial for clinical translation of MSC-EVs and will ultimately provide T2D patients with an effective and safe treatment option.

**Supplementary Information:**

The online version contains supplementary material available at 10.1186/s13287-026-04991-w.

## Introduction

**Fig. 1:**
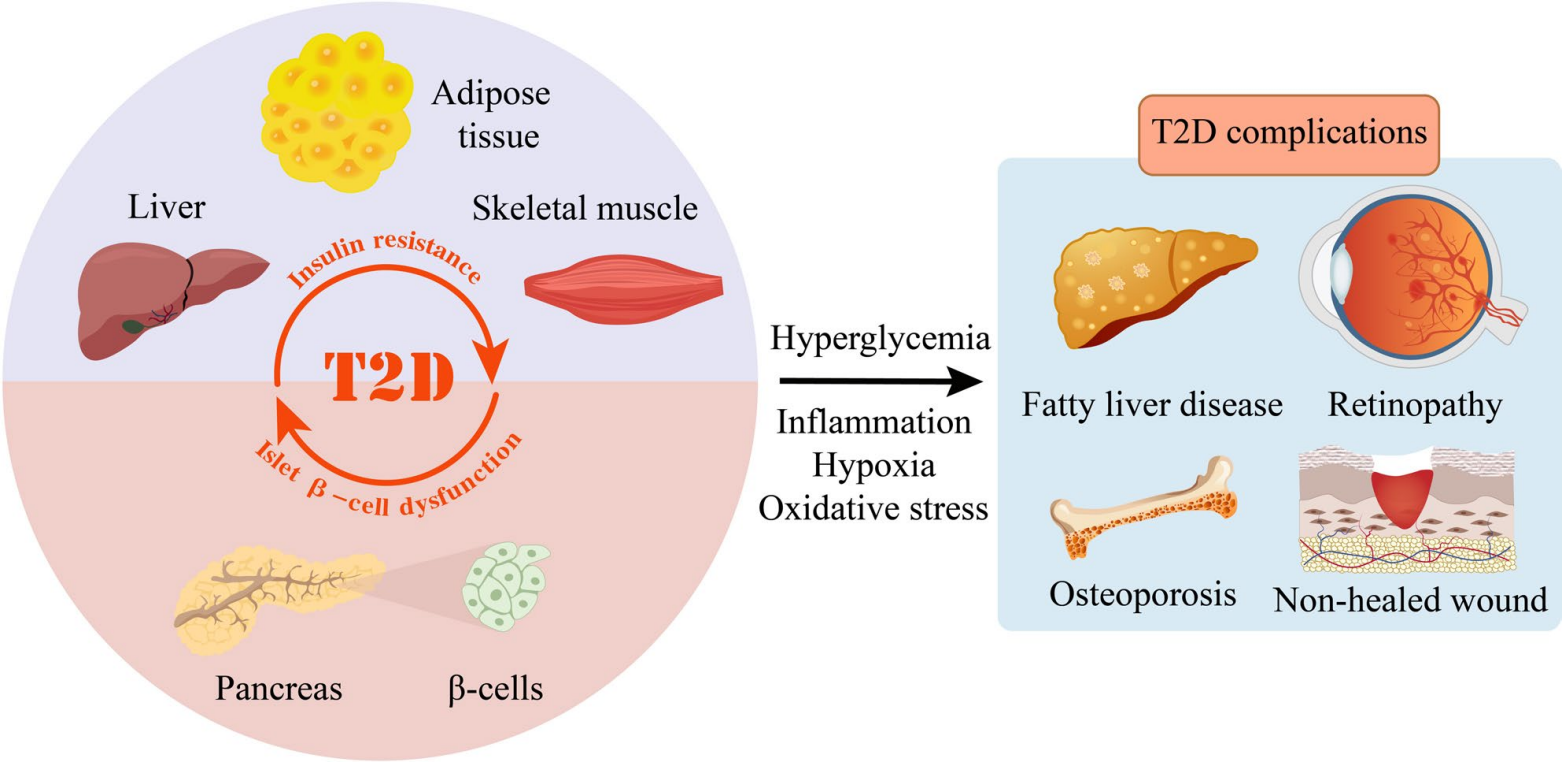
Schematic overview of the pathogenic cascade from type 2 diabetes (T2D) to systemic complications. T2D is primarily characterized by insulin resistance in peripheral tissues such as adipose tissue, liver, and skeletal muscle, as well as dysfunction of pancreatic islet β-cells. Insulin resistance and islet β-cell dysfunction reciprocally aggravate each other and synergistically exacerbate hyperglycemia. Hyperglycemia triggers a series of pathological processes, including inflammation, hypoxia, and oxidative stress. These pathological changes contribute to the development of multiple complications, including liver steatosis, non-healed wounds, retinopathy and bone loss

Type 2 diabetes (T2D) is a rapidly growing metabolic disorder that presents significant challenges to global public health owing to its intricate pathophysiological mechanisms and extensive complications. T2D arises from inadequate utilization or decreased efficiency of insulin [1]. The global prevalence of T2D is gradually increasing [2], which is estimated to reach 12.2% (783.2 million people worldwide) by 2045 [3]. The development of T2D is closely linked to insulin resistance and β-cell dysfunction [4], in which insulin resistance is pivotal in T2D pathogenesis [5], while β-cell dysfunction leads to abnormal insulin secretion [6]. In T2D, persistent hyperglycemic environment can trigger inflammation, localized hypoxia, and increased oxidative stress. These conditions contribute to various complications [7, 8], such as wound healing impairment, retinopathy, and osteoporosis (Fig. 1) [9–12]. Current treatment approaches of T2D include pharmacotherapy [13], exercise, and dietary management [14], which alleviate symptoms of the metabolic disorder but cannot reverse or fundamentally cure T2D and its associated multi-organ dysfunction [15]. There is an urgent need for the development of novel therapeutic strategies to enhance patient quality of life and slow disease progression for T2D.

Mesenchymal stem cells (MSCs) are characterized by plastic adhesion properties, self-renewal, immunomodulation, and multiple differentiation potentials [16]. They can be derived from a wide array of tissues and are extensively tested in therapeutic applications [17]. Notably, our research group has conducted a pilot clinical trial showing that MSC treatment for T2D patients has beneficial effects yet certain limitations, with its efficacy being influenced by the disease’s severity [18]. Therefore, it is essential to further elucidate the mechanisms by which MSCs effectively treat T2D and to optimize treatment strategies for severe cases based on these insights. In this context, our studies and others’ have further illustrated that MSCs exert regulatory and therapeutic effects on T2D metabolism by releasing extracellular vesicles (EVs) [19], nano-sized particles secreted by cells, enclosed by a lipid bilayer [20], present in biological fluids and involved in intercellular communication in physiological and pathological processes [21]. MSC-derived EVs (MSC-EVs) have shown immense potential in T2D treatment [22, 23]. Further investigation into the therapeutic targets of MSC-EVs and the development of targeted engineering strategies are expected to provide new methodologies for stem cell-based therapies for T2D [24, 25].

Recent studies highlight the promising role of MSCs in treating T2D through EV-mediated paracrine effects [26, 27]. MSC-EVs have been shown to improve [28, 29] or even reverse [30] insulin resistance in T2D animal models, thereby enhancing the glucose metabolism. Additionally, research suggests that MSC-EVs achieve therapeutic effects by promoting islet cell proliferation [31] and alleviating β-cell destruction [30]. They also could reduce oxidative stress and inhibit inflammation in T2D [32, 33] which show great potential for tissue repair [34]. MSC-EVs deliver multiple active substances to modulate recipient cell behaviors [35], and their biodistribution and activity can be optimized through appropriate engineering modifications [36]. This review summarizes the roles and mechanisms of MSC-EVs from various sources in treating T2D, exploring their potential applications in T2D and its associated complications. We have also discussed the current limitations and future directions of this field based on the cutting-edge interdisciplinary knowledge.

## Characteristics of MSC-EVs

In the 1970s, Friedenstein et al. discovered a type of cell that could differentiate and adhere to plastic surfaces under culture conditions [37]. In 1991, Caplan named these cells mesenchymal stem cells [38]. By July 2024, 1290 clinical trials related to MSCs have been registered on ClinicalTrials.gov. Currently, there are 62 MSC therapies undergoing clinical trials in China. MSCs and related products are extensively applied in the treatment of immune, inflammatory, and metabolic diseases [27]. As multipotent stem cells, MSCs possess not only the potential for multilineage differentiation [38], including mesodermal, endodermal and neuroectodermal lineages [39], but also immunomodulatory and metabolic regulatory potential. Immunomodulatory properties of MSCs are evidenced by interactions with myeloid and lymphoid cells, including T cells, B cells, and antigen-presenting cells [40]. Their metabolic regulatory capabilities are tied to mitochondrial metabolic processes [41]. These characteristics make MSCs a valuable resource in the development of new therapeutic strategies for a variety of diseases.

**Fig. 2:**
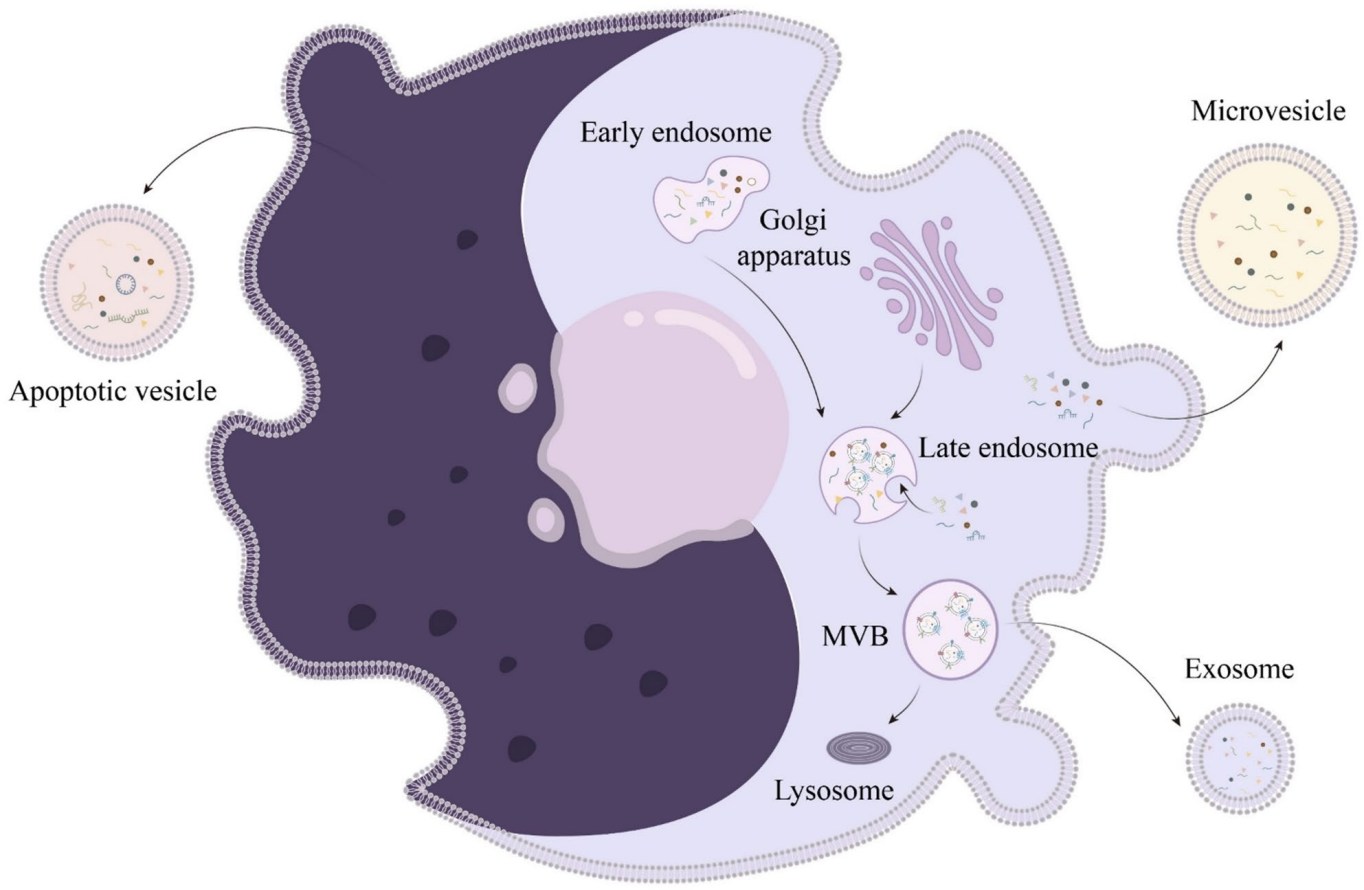
Classification and biogenesis of extracellular vesicles (EVs). EVs are typically categorized into three main types: exosomes, microvesicles, and apoptotic vesicles. Exosomes are generated through the endocytic pathway, originating from the early endosome, which matures into a late endosome and subsequently forms multivesicular bodies (MVBs). These MVBs fuse with the cell membrane to release exosomes. Microvesicles are directly produced from the cell membrane through a process involving membrane budding and shedding. Apoptotic vesicles are released during the apoptosis process when the cell membrane blebs and forms vesicles containing cellular components

Clinical trials have demonstrated that the core therapeutic goals of MSC therapy include improving glycemic control (e.g., reducing HbA1c and insulin requirements) and preserving islet β-cell function (e.g., increasing C-peptide levels) [42]. Notably, emerging evidence suggests that these therapeutic effects may be at least partially attributed to the paracrine actions of MSC-derived extracellular vesicles, as EVs have been established as key mediators of intercellular communication and tissue repair [27]. Therefore, it is noteworthy that the therapeutic effects of MSCs are closely associated with the EVs they secrete. Based on the biogenesis and sizes, EVs are typically classified into three subtypes: exosomes originating from the endosomal pathway, microvesicles derived from plasma membrane budding, and apoptotic vesicles (traditionally called apoptotic bodies) produced during apoptosis [43, 44]. These nanoscale vesicles exhibit a lipid bilayer structure enriched with hydrophilic proteins, nucleic acids, etc. (Fig. 2) [45]. EVs demonstrate multifunctional biological roles, serving as direct signaling mediators to recipient cells [46] and facilitating the intercellular transfer of bioactive cargoes [47]. EVs play pivotal roles in mitigating inflammatory and metabolic disorders, which participate in diverse pathophysiological conditions [48, 49]. EVs show remarkable therapeutic potential [21], particularly MSC-EVs that demonstrate therapeutic advantages by preventing potential immunogenicity [50] and ectopic tissue formation [51] issues associated with direct MSC transplantation.

MSCs have diverse sources and can be isolated from various tissues, including the umbilical cord, the bone marrow, and the adipose tissue [52, 53], which demonstrate good histocompatibility and stability [54]. MSC-EVs inherit excellent accessibility from MSCs and are equipped many advantages of EVs, such as easy transfer, potential to recognize recipient cells, the ability to cross biological barriers [55], and the feasibility to store at low temperatures [56] to prevent content degradation. In addition, MSC-EVs show limited immunogenicity and are non-tumorigenic, which play an important role in disease treatment by delivering multiple bioactive factors or providing drug delivery [57]. However, due to insufficient targeting or limited cargo content, their therapeutic effects are suboptimal, thus engineered MSC-EVs have gradually become a research focus. Methods for engineering MSC-EVs include optimizing MSCs to achieve higher yield and quality of EVs [58], loading various contents into MSC-EVs [11], or developing sustained-release systems [59] to enhance therapeutic effectiveness of MSC-EVs. These characteristics provide MSC-EVs with important potential and promising applications in treating a broad spectrum of diseases, including T2D. A recent clinical trial of MSC-EVs has been initiated (NCT07144241), marking the first registered clinical trial specifically investigating MSC-EVs in T2D, signifying the transition of MSC-EV therapy from preclinical research toward clinical translation.

## Mechanisms and prospects of natural MSC-EVs in treating T2D and its complications

**Fig. 3:**
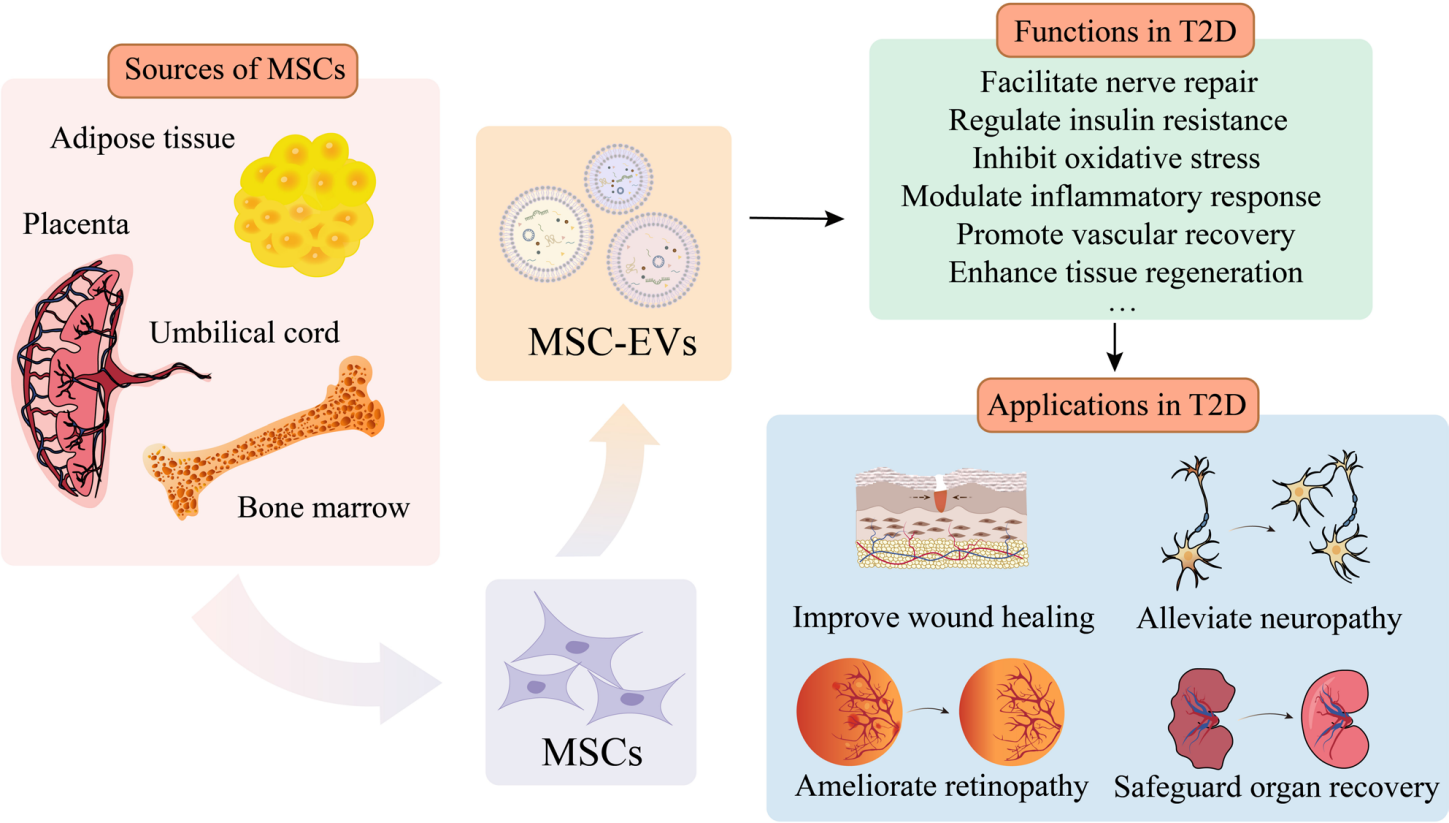
Mesenchymal stem cell-derived extracellular vesicles (MSC-EVs) from various tissue sources and their functions in type 2 diabetes (T2D). MSCs can be isolated from multiple tissues, including the adipose tissue, the placenta, the umbilical cord, and the bone marrow. These MSCs secrete EVs that play diverse roles in T2D management. The functions of MSC-EVs in T2D include facilitating nerve repair, regulating insulin resistance, inhibiting oxidative stress, modulating inflammatory responses, promoting vascular recovery, and enhancing tissue regeneration. These capabilities translate into potential therapeutic applications for T2D and its complications, such as improving wound healing, alleviating neuropathy, ameliorating retinopathy, and safeguarding organ recovery

Natural EVs derived from MSCs show potential therapeutic value and have become a focal point in current diabetes research (Fig. 3; Supplementary Table 1).

### EVs released in normal conditions

#### Human umbilical cord mesenchymal stem cell-derived EVs (UCMSC-EVs)

UCMSCs are easily obtainable and have minimal ethical constraints. UCMSC-EVs demonstrate vital roles in treating T2D and its complications [60], which positively affect various aspects of diabetes pathology through their bioactive molecules [60–62]. Firstly, UCMSC-EVs play an important role in regulating insulin resistance. UCMSC-EVs can reduce homeostatic model assessment for insulin resistance (HOMA-IR) and suppress blood glucose peaks [28], indexes for diabetic management, while enhancing the expression of phosphorylated insulin receptor substrate 1 (IRS-1) and protein kinase B (AKT), downstream signaling of insulin receptor, thus restoring insulin sensitivity [30]. This effect is vital for disrupting the vicious cycle between insulin resistance and hyperglycemia. Secondly, UCMSC-EVs inhibit oxidative stress in the hyperglycemic microenvironment of T2D, alleviating cellular injury and tissue dysfunction [63]. Specifically, UCMSC-EVs deliver the neuronally expressed developmentally downregulated 4 (NEDD4) to diabetic retinal cells, which promotes ubiquitination and degradation of phosphatase and tensin homologue (PTEN) and activates the AKT/nuclear factor erythroid 2-related factor 2 (NRF2) signaling to enhance the antioxidant capacity [64]. Additionally, the miR-5068 and miR-10,228 contained within UCMSC-EVs can target the hypoxia-inducing factor (HIF)-1α/enhancer of zeste homolog 2 (EZH2)/peroxisome proliferator-activated receptor-gamma coactivator (PGC)-1α pathway to improve the antioxidant and anti-apoptotic capacity of retinal cells in T2D mice [11]. These mechanisms provide new therapeutic targets to protect tissues like the retina, which are susceptible to oxidative stress damage. Thirdly, UCMSC-EVs effectively regulate inflammatory responses in T2D. T2D patients are usually kept in a state of low-grade chronic inflammation, where abnormal release of inflammatory factors including interleukin (IL)-1β, IL-6, and tumor necrosis factor (TNF)-α not only exacerbates insulin resistance but also aggravates diabetic complications. UCMSC-EVs suppress excessive activation of inflammatory responses by regulating the release of these cytokines, thus reducing chronic inflammatory tissue damages [65]. Fourthly, UCMSC-EVs emerge as pivotal mediators of vascular functional recovery and angiogenesis. Microvascular lesions and vascular dysfunction are severe complications of T2D, affecting blood perfusion and nutrient supply to multiple tissues/organs. Research shows that UCMSC-EVs significantly promote endothelial cell (EC) proliferation, possibly improving microvascular permeability and promoting angiogenesis through the transforming growth factor (TGF)-β1 signaling [62]. Wei et al. demonstrated that UCMSC-EVs target the PTEN/AKT/HIF-1α/vascular endothelial growth factor (VEGF) pathway to enhance proliferation, migration, and tube formation of ECs in the high glucose condition, facilitating wound-site angiogenesis in T2D animals, thereby providing essential vascular support for tissue repair and regeneration [60]. Lastly, UCMSC-EVs also show potential in neural protection and repair. T2D patients usually suffer from impairments in the nervous system, such as peripheral neuropathy and autonomic neuropathy, which severely affect patients’ life quality. UCMSC-EVs carry neurotrophic factors, such as the brain-derived neurotrophic factor (BDNF) [66], which promote regeneration and protection of neuronal cells, improve neural conductivity, and delay neuropathy progression, holding potential clinical value for alleviating neurological symptoms and enhancing self-care ability in T2D patients. A thorough understanding of the more in-depth mechanisms underlying UCMSC-EV function is crucial for developing effective therapeutic strategies, which will provide a solid foundation for future diabetic treatment.

#### Bone marrow mesenchymal stem cell-derived EVs (BMMSC-EVs)

**Fig. 4:**
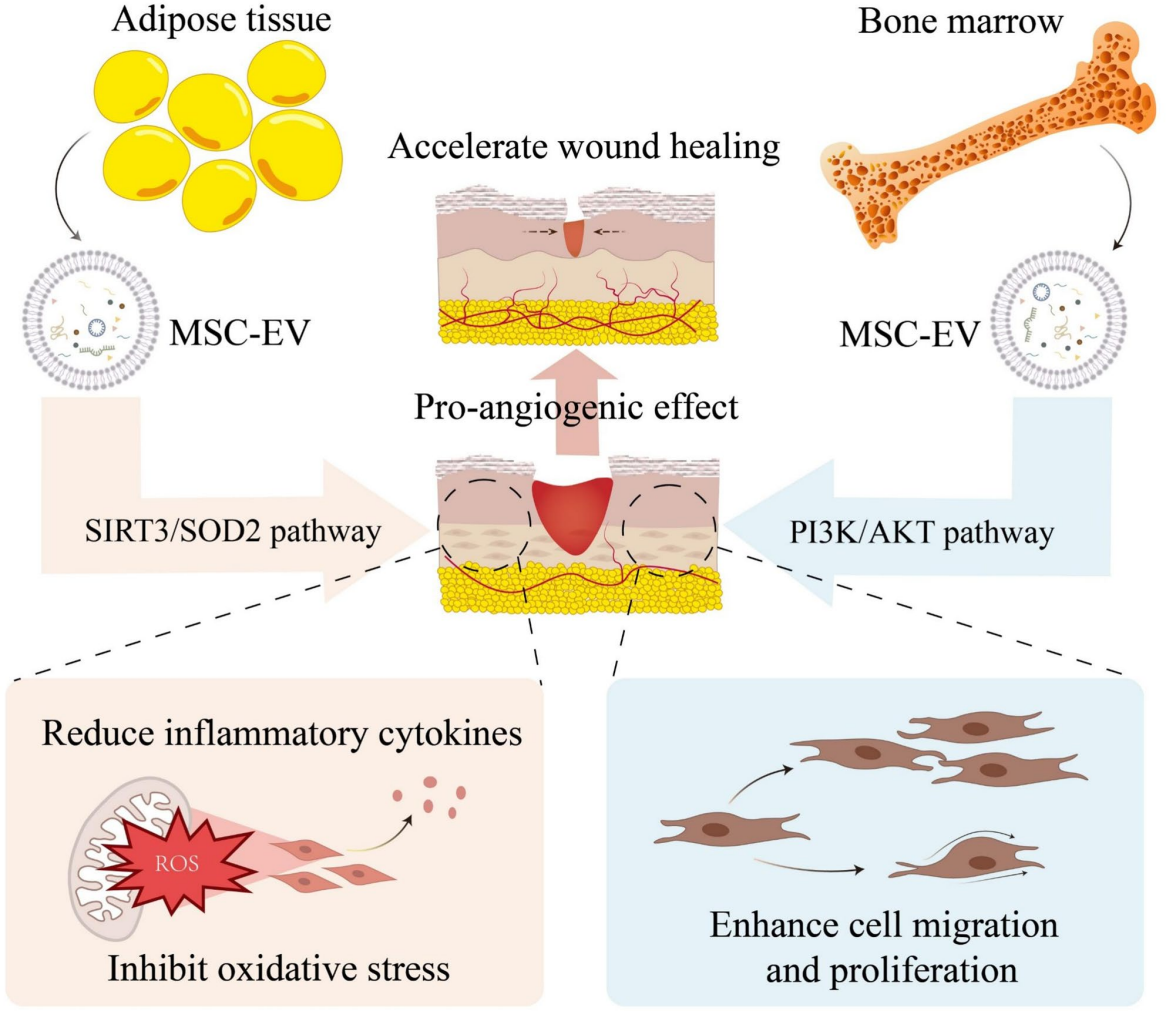
Distinct mechanisms of adipose mesenchymal stem cell-derived extracellular vesicles (ADMSC-EVs) and bone marrow mesenchymal stem cell-derived extracellular vesicles (BMMSC-EVs) in promoting wound healing. ADMSC-EVs accelerate wound healing by inhibiting oxidative stress and reducing inflammatory cytokines via the Sirtuin 3 (SIRT3)/Superoxide dismutase 2 (SOD2) pathway. On the other hand, BMMSC-EVs enhance cell migration and proliferation, and exert a pro-angiogenic effect through the Phosphatidylinositol 3-kinase (PI3K)/AKT pathway

BMMSCs are playing increasingly crucial roles in the field of regenerative medicine due to the multilineage differentiation potential and immune regulation capabilities [67]. BMMSC-EVs inherit the regenerative and immunomodulatory characteristics of their parent cells, showing broad application potential in treating T2D and its complications [68]. Similar to UCMSC-EVs, BMMSC-EVs can enhance insulin sensitivity, while they function through different mechanisms [69]. BMMSC-EVs promote the expression and transport of glucose transporter 4 (GLUT4) by activating the phosphoinositide 3-kinase (PI3K)/AKT signaling pathway, thereby improving the efficiency of glucose uptake and utilization, offering direct therapeutic benefits for correcting glucose metabolic disorders in T2D individuals [29]. BMMSC-EVs also show good effects in inhibiting inflammation in the hyperglycemic environment of T2D [70]. BMMSC-EVs can reduce levels of pro-inflammatory cytokines TNF-α and IL-1β in the serum, modulate macrophage polarization from the M1 to M2 phenotype, decrease the number of activated macrophages, and target the Toll-like receptor 4 (TLR4)/ nuclear factor Kappa B (NF-κB) signaling *via* microRNAs to alleviate peripheral nerve inflammation and dysfunction in T2D mice [71]. Moreover, BMMSC-EVs have notable effects in promoting diabetic tissue regeneration. In diabetic mouse models, BMMSC-EVs are internalized by recipient fibroblasts, which then upregulate PTEN to suppress excessive activation of the PI3K/AKT signaling pathway. This effect might also be related to fibroblastic insulin responses, promoting fibroblast proliferation and migration while upregulating angiogenic factors. Through these mechanisms, BMMSC-EVs accelerate the wound healing process in diabetic mice, providing support for tissue regeneration and repair [72]. Furthermore, BMMSC-EVs demonstrate significant potential in the field of neurological repair. Clinical studies have indicated that glycemic control alone is insufficient to effectively delay or reverse the progression of peripheral neuropathy associated with T2D, underscoring the urgent need for nerve-targeted repair strategies [73]. In this context, BMMSC-EVs present a promising alternative therapeutic approach. Experimental research has shown that BMMSC-EVs can effectively ameliorate peripheral neuropathy in animal models of T2D by increasing intraepidermal nerve fiber density and promoting axonal myelination [71]. This strategy offers new hope for achieving fundamental improvement in neurological function among T2D patients. In summary, BMMSC-EVs demonstrate broad application prospects in the treatment of T2D and its various complications through multiple mechanisms, providing important experimental evidence for the development of novel therapeutic strategies (Fig. 4).

#### Adipose mesenchymal stem cell-derived EVs (ADMSC-EVs)

As previously mentioned, BMMSC-EVs play a significant role in promoting wound healing in T2D animal models by enhancing cell proliferation and migration. Similarly, ADMSC-EVs have demonstrated substantial therapeutic potential in the wound healing process induced by T2D (Fig. 4). These effects are closely associated with their ability to promote angiogenesis and improve cellular function in vivo. These findings not only open new avenues for future disease treatment but also emphasize the importance of selecting appropriate stem cell sources when developing EV-based therapies [74]. Recent studies have further revealed that ADMSC-EVs can alleviate local oxidative stress and suppress the secretion of inflammatory cytokines in diabetic wounds by modulating the sirtuin 3 (SIRT3)/superoxide dismutase 2 (SOD2) signaling pathway, thereby enhancing periwound vascularization and accelerating the healing process [32]. This mechanism holds important clinical implications for the treatment of chronic skin ulcers, which are commonly observed in T2D patients. Beyond cutaneous wound repair, ADMSC-EVs also show promise in the treatment of diabetic nephropathy (DN), a severe complication of T2D with a complex pathogenesis and limited therapeutic options. In T2D mouse models, ADMSC-EVs treatment significantly reduced renal injury markers such as urinary protein, serum creatinine, and blood urea nitrogen, and ameliorated pathological changes in renal tissues. Mechanistic studies indicate that ADMSC-EVs deliver miR-26a-5p to target TLR4, thereby regulating the NF-κB/VEGFA signaling pathway, suppressing inflammatory responses and apoptosis, and protecting glomerular podocytes from high glucose-induced damage [75]. Furthermore, ADMSC-EVs can also inhibit PDK4 via miR-15b-5p, downregulate VEGFA expression, and further reduce apoptosis and inflammation, exerting protective effects in high glucose-induced podocyte injury models [76]. Taken together, ADMSC-EVs exhibit pleiotropic benefits against T2D pathologies, positioning them as a novel class of therapeutics for diabetic complications [77].

#### Placenta mesenchymal stem cell-derived EVs (PMSC-EVs)

Placenta derived-MSCs (PMSCs) have demonstrated significant potential in the treatment of diabetes and its complications [78]. Studies have shown that PMSCs can improve insulin sensitivity and restore glucose homeostasis by activating the PI3K-Akt signaling pathway, offering a novel therapeutic strategy for obesity-associated T2D [78]. In recent years, EVs derived from PMSCs have gained increasing attention as a cell-free therapeutic approach. Experimental studies have confirmed that local administration of PMSC-EVs significantly promotes the healing of full-thickness skin wounds in type 2 diabetic mice, accelerating wound closure, reducing scar width, and facilitating organized collagen deposition. Mechanistically, PMSC-EVs are enriched with miR-145-5p, which targets cyclin-dependent kinase inhibitor 1 A (CDKN1A) and activates the extracellular signal-regulated kinase (ERK) and protein kinase B (AKT) signaling pathway, thereby enhancing cell proliferation, migration, and anti-apoptotic activity, collectively driving the repair of diabetic refractory wounds [79]. However, the clinical application of PMSC-EVs faces considerable challenges. Studies have indicated that the antioxidant activity in PMSC-conditioned medium primarily resides in non-EV components, suggesting that EVs alone may not fully replicate the therapeutic functions of their parental cells [80]. Furthermore, critical parameters such as EV stability, in vivo delivery efficiency, optimal dosage, and treatment timing remain to be systematically elucidated. In conclusion, PMSC-EVs exhibit promising potential in ameliorating insulin resistance and promoting wound healing via the delivery of specific miRNAs and regulation of key signaling pathways. Despite challenges such as incomplete functional composition and standardization in preparation, continued investigation into their mechanisms of action and delivery systems may position PMSC-EVs as an effective cell-free strategy for managing diabetic complications.

### Apoptotic vesicles (ApoVs)

**Fig. 5:**
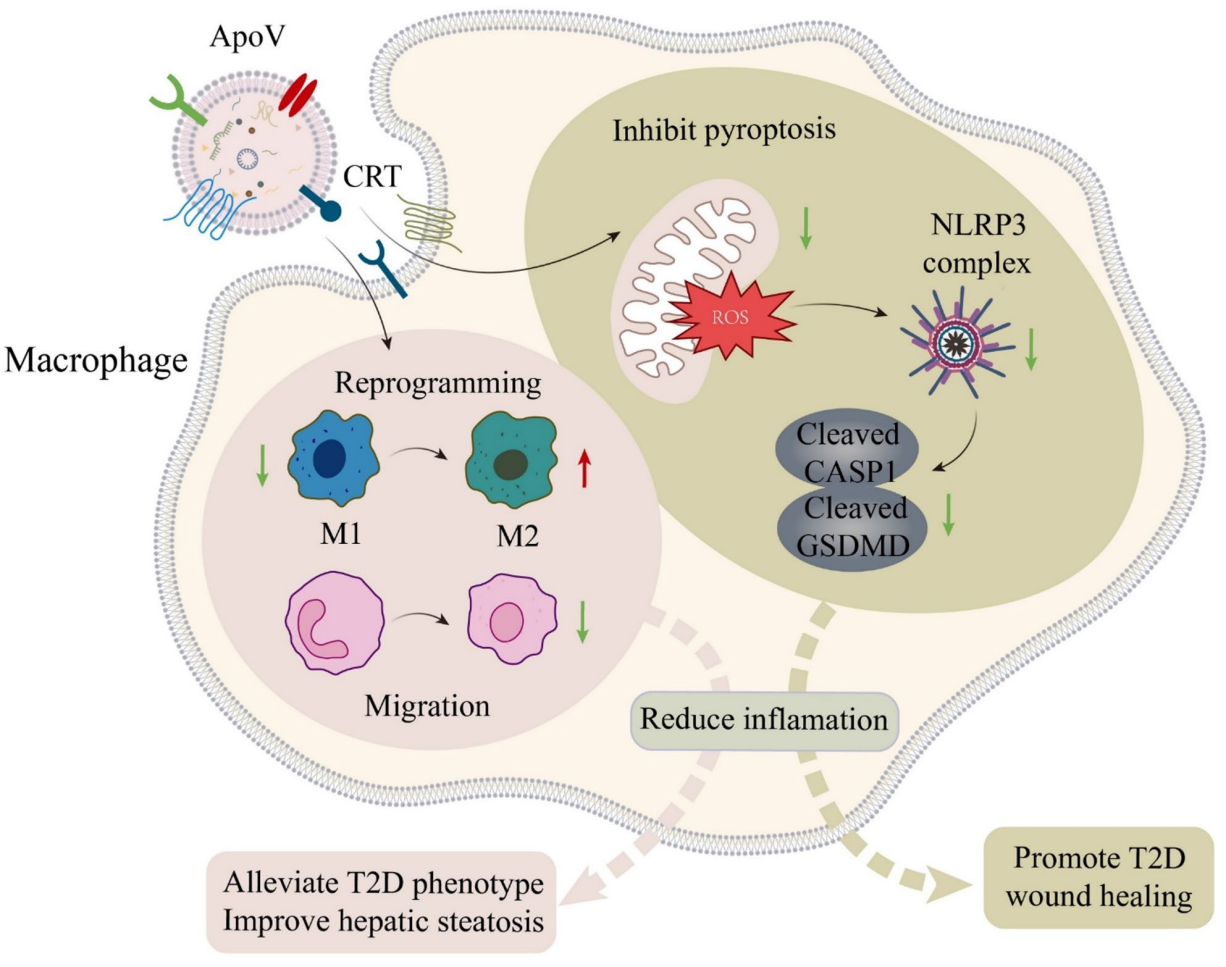
Mechanisms of apoptotic vesicles (apoVs) in alleviating type 2 diabetes (T2D). ApoVs play a crucial role in mitigating T2D progression through multiple mechanisms. They are endocytosed by macrophages *via* calreticulin (CRT) and promote macrophage reprogramming from the pro-inflammatory M1 phenotype to the anti-inflammatory M2 phenotype, which reduces inflammation and enhances tissue repair. ApoVs also inhibit pyroptosis of macrophages by decreasing reactive oxygen species (ROS) production and suppressing the NOD-, LRR- and pyrin domain-containing protein 3 (NLRP3) inflammasome complex, thereby reducing the cleavage of Caspase 1 (CASP1) and gasdermin D (GSDMD). These actions collectively alleviate insulin resistance, improve hepatic steatosis, and promote wound healing in T2D

ApoVs are EVs released by MSCs and other cells during apoptosis, which occurs under various physiological and pathological conditions and participates in multiple biological functions, including immune regulation, tissue/organ homeostasis maintenance, and regulation of disease progression [81]. Studies indicate that under equal MSC culture number, the yield of apoVs exceeds that of exosomes by more than tenfold, and that compared to exosomes, apoVs possess a unique protein expression profile [82, 83]. Our research using proteomic analysis has revealed that apoVs carry abundant functional proteins closely related to regulation of cell behaviors, signal transduction, immune responses, and metabolic processes [83]. The administration of MSC-derived apoVs (MSC-apoVs) has been confirmed as an effective biological therapy for multiple conditions, such as immune diseases, osteoporosis, skin damages, and tumors [81]. Notably, MSCs are known to undergo apoptosis and produce apoVs after transplantation, and MSC-apoVs exert positive effects on metabolic abnormalities in T2D *via* multiple mechanisms. Zheng et al.. have shown that MSC-apoVs improve glucose tolerance, alleviate insulin resistance, and reduce hepatic steatosis in T2D after systemic infusion [83]. With engraftment in the liver, MSC-apoVs recognize hepatic macrophages in treating T2D by surface-exposed calreticulin (CRT), promoting their polarization toward an anti-inflammatory phenotype and suppressing liver infiltration of bone marrow-derived monocytes [83]. With regard to local application, implantation of MSC-apoVs in the T2D unhealing wounds reduces skin macrophage inflammation through inhibiting activation of the NOD-, LRR- and pyrin domain-containing protein 3 (NLRP3) inflammasome, which supresses pyroptosis, an inflammatory form of cell death. Thus, MSC-apoVs decreases inflammatory cytokine production of macrophages and significantly improves healing of cutaneous defects, promoting tissue repair in T2D [84]. Additional experiments have revealed that MSC-apoVs reduce macrophage reactive oxygen species (ROS), contributing to the alleviation of T2D-associated pathological conditions [84]. In brief summary, MSC-apoVs exhibit diverse therapeutic mechanisms in T2D and related complications (Fig. 5). Future studies will further elucidate the specific molecular mechanisms of apoVs and assess their efficacy and safety in the clinical practice.

### EVs produced by MSCs from the T2D condition (T2D-MSC-EVs)

**Fig. 6:**
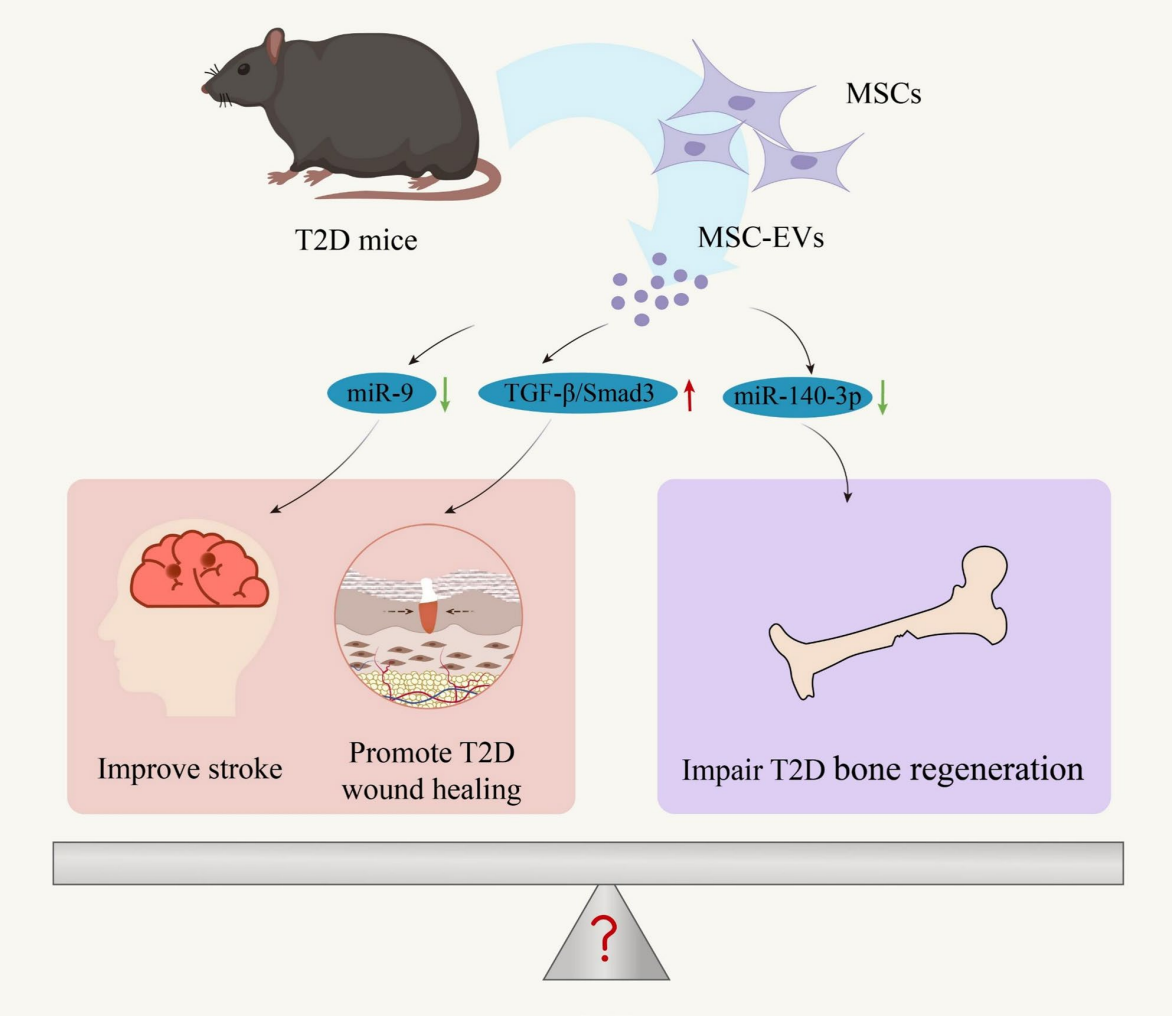
Dual roles of type 2 diabetes (T2D)-sourced mesenchymal stem cell-derived extracellular vesicles (MSC-EVs). MSC-EVs isolated from T2D mice exhibit dual effects: they promote neural repair and wound healing by modulating pathways, such as miR-9 and Transforming growth factor (TGF)-β/Smad3; conversely, they may also negatively impact bone regeneration by regulating miR-140-3p

Compared to MSC-EVs from normal and apoptotic states, studies focusing on MSC-EVs derived from diseased states are relatively limited, and their potential pathological regulatory roles and detailed mechanisms require further investigation. Some research has uncovered a potentially negative role of T2D-MSC-EVs in bone defect healing. T2D-MSC-EVs are demonstrated to carry specific microRNAs, such as miR-140-3p, which are expressed at lower levels compared to healthy MSC-EVs, impairing the osteogenic differentiation capacity of recipient BMMSCs after treatment. In contrast, implantation of EVs enriched with miR-140-3p into damaged bone sites of T2D animal models accelerates bone regeneration. This suggests that modulating microRNA expression in T2D-MSC-EVs may help to enhance bone healing in T2D, providing novel targets for treating T2D-associated bone diseases [12]. Notably, several studies have reported beneficial functions of T2D-MSC-EVs in ameliorating T2D complications, showing autologous therapeutic potential and gradually becoming a research interest. As a major risk factor for stroke, T2D not only increases stroke incidence but also worsens post-stroke recovery. Studies have discovered that compared to EVs secreted by BMMSCs under non-diabetic conditions, EVs from BMMSCs in the T2D state (T2D-BMMSC-EVs) possess unique microRNA expression profiles and biological functions. In this regard, decreased miR-9 expression in T2D-MSC-EVs correlates closely with improved neurological function, enhanced white matter remodeling, and strengthened anti-inflammatory effects. Treating stroke T2D rats with T2D-MSC-EVs significantly promote neurological recovery, restore blood-brain barrier integrity, enhance white matter remodeling, increase axonal growth of cortical neurons, and effectively boost anti-inflammatory responses. This discovery offers a new therapeutic approach for post-stroke rehabilitation in T2D patients [85]. In terms of chronic wounds, Hsu et al.. revealed a therapeutic strategy using EVs secreted by ADMSCs from T2D mice (T2D-ADMSC-EVs) to promote wound healing. This research indicates that these T2D-ADMSC-EVs significantly promote secretion of TGF-β1 and activate the TGF-β/Smad3 signaling, a critical pathway regulating fibroblast proliferation and activation, thereby initiating wound healing. Utilizing T2D-ADMSC-EVs as a treatment may also reduce the risk of immune rejection and enhance feasibility of autologous therapy using stem cell derivatives, holding potential clinical value for T2D applications [86]. In brief summary, the diverse mechanisms of T2D-MSC-EVs revealed by these studies provide controversial yet fresh perspectives for treatment of T2D complications, which inspire open questions as when and which molecular targets function to exert expected effects (Fig. 6).

Taken together, EVs from various sources and types show broad therapeutic potential in treating T2D and its complications. Future studies will further elucidate their precise molecular mechanisms and clinical efficacy, as well as clarifying whether MSC-EVs derived from different tissues possess distinct characteristics and advantages in treating T2D and specific complications, aiming to offer new hope for T2D therapy.

## Engineered MSC-EVs: an emerging aspect in the treatment of T2D and its complications

In the treatment of T2D and its complications, engineered MSC-EVs are emerging as promising approaches that may change the current therapeutic landscape (Supplementary Table 1). With the advancing research, both biologists and biomedical engineers are gradually gaining insight into their immense application potential and profound translational significance. Metaphorically, natural MSC-EVs are just like a talented dancer yet restricted by a limited stage, bringing hope for the treatment of T2D and its complications. Nonetheless, “stage limitations” such as the production yield, the quality, and the heterogeneity prevent this natural therapeutic modality from fully flourishing in widespread clinical application. Consequently, engineered MSC-EVs have emerged, akin to providing the dancer with an entirely new and expanded stage, utilizing the remarkable “toolboxes” of genetic engineering and multiple biotechnologies to optimize MSC-EV production processes, thereby enhancing the yield and quality. More encouragingly, constructing efficient delivery and functional systems from a biomaterial perspective can further improve the stability and therapeutic efficacy of MSC-EVs.

### Enhancing EV yield and quality by optimizing MSC culture conditions

**Fig. 7:**
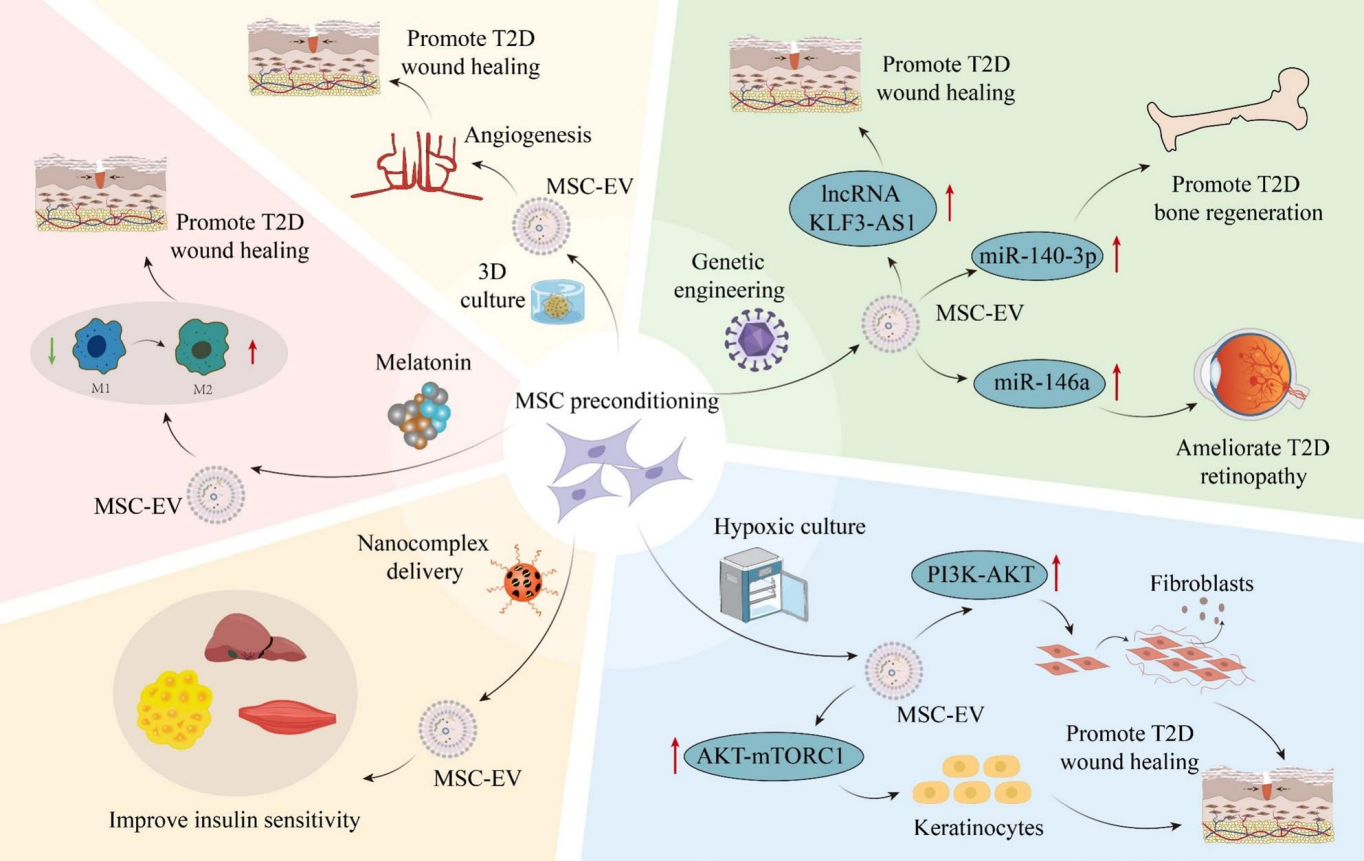
Enhancing extracellular vesicle (EV) yield and quality by optimizing mesenchymal stem cell (MSC) culture conditions. Engineering MSC-EVs through preconditioning strategies, such as hypoxic culture, melatonin pretreatment, three-dimensional (3D) culture, genetic engineering, and nanocomplex delivery enhances their therapeutic efficacy in type 2 diabetes (T2D). These approaches optimize EV yield and quality, improve insulin sensitivity, promote wound healing, and support bone regeneration by modulating specific molecular pathways

MSC-EVs exert therapeutic effects in various disease models, and their efficacy is closely related to the statuses of MSCs [58]. MSC statuses are influenced by numerous microenvironmental factors or molecular interventions, which can be harnessed to substantially improve EV yield and quality, such as the hypoxic preconditioning, hormonal stimulation, signaling factor modulation, three-dimensional (3D) culture techniques, and lentiviral transfection [87]. These conditions can also enhance the function of MSC-EVs in tissue repair and immunomodulation (Fig. 7) [88]. ADMSC-EVs cultured under hypoxia precisely regulate their microRNA expression profiles and activate the PI3K/AKT signaling pathway, which boost fibroblast survival and proliferation in skin wounds, promote production of extracellular matrix (ECM) and growth factors, thus accelerating wound healing in T2D models [89]. Similarly, hypoxia-preconditioned BMMSC-EVs exhibit remarkable therapeutic potential through the upregulated miR-4645-5p activating autophagy *via* complex molecular regulation, offering benefits for wound healing in T2D mice [90]. The strategic application of melatonin preconditioning also endows MSC-EVs with enhanced therapeutic capacity by modulating the PTEN/AKT pathway, which increase the macrophage M2 to M1 polarization ratio, suppressing inflammatory responses and promoting wound healing in diabetic rats [91]. Furthermore, an aptamer-hallmarked nanocomposite-mediated miR-29b-3p regulation technology has been established to optimize MSC-EV function. Precise delivery of anti-miR-29b-3p oligonucleotides into BMMSCs *via* this nanotechnology successfully reduces miR-29b-3p levels in the secreted EVs, which activate insulin signaling pathways and significantly improve insulin resistance in aged mice, expanding therapeutic horizons for metabolic diseases [92]. 3D culture technology, by simulating the mesenchymal condensation phenomenon during development to construct stem cell aggregates, significantly optimizes the function of MSC-EVs. On one hand, this strategy activates the Notch signaling pathway, enhancing the pro-angiogenic capacity of MSC-EVs and specifically inducing the formation of CD31/EMCN double-positive vessels, thereby providing robust support for the healing of T2D skin defects [93]. On the other hand, EVs released by stem cell aggregates from human exfoliated deciduous teeth (SHED) effectively recruit the Gli1^+^ cell subpopulation from hair follicle stem cells to the wound edge, enhancing their differentiation into epidermal cells and accelerating re-epithelialization, thus promoting diabetic wound healing [94]. Lentiviral transfection of various signaling molecules further empowers MSC-derived EVs to elevate their therapeutic efficacy across multiple T2D complications, including bone defects [12], unhealed cutaneous wounds [34, 95], diabetic limb ischemia [96], and peripheral neuropathy [97]. Furthermore, preconditioning MSCs through directed differentiation has opened a new dimension for enhancing the therapeutic efficacy of their EVs. Studies have shown that EVs derived from MSCs differentiated into insulin-producing cells can modulate naïve MSCs to become insulin-secreting, offering a novel strategy for diabetes treatment [98]. These strategies not only strengthen the therapeutic outcomes of MSC-EVs but also provide safer and more effective therapeutic options, demonstrating broad potential for application. Future research should further elucidate specific parameters for optimized MSC culture conditions, such as the precise oxygen pressure range for hypoxic induction and potential adverse effects beyond certain thresholds on MSC-EVs, as well as exploring integrating artificial intelligence (AI) and big data to determine comprehensive condition combinations aiming to synergistically enhance MSC-EV yield and functionality. Future studies also need to clarify the dialectical relationships between the yield and function MSC-EVs, particularly, whether and how optimization of MSC culture condition differentially affects the production and functional performance of MSC-EVs, and how to balance the quantity and quality of MSC-EVs to better promote clinical applications. It is recommended to adopt standardized MSC culture, EV extraction, and detection methodologies to conduct comparative studies under uniform conditions. Moreover, employing advanced imaging and multi-omics technologies to achieve high-precision and high-throughput analysis will facilitate addressing these aforementioned issues.

### Enhancing therapeutic functions of EVs by modifying their cargo molecules

**Fig. 8:**
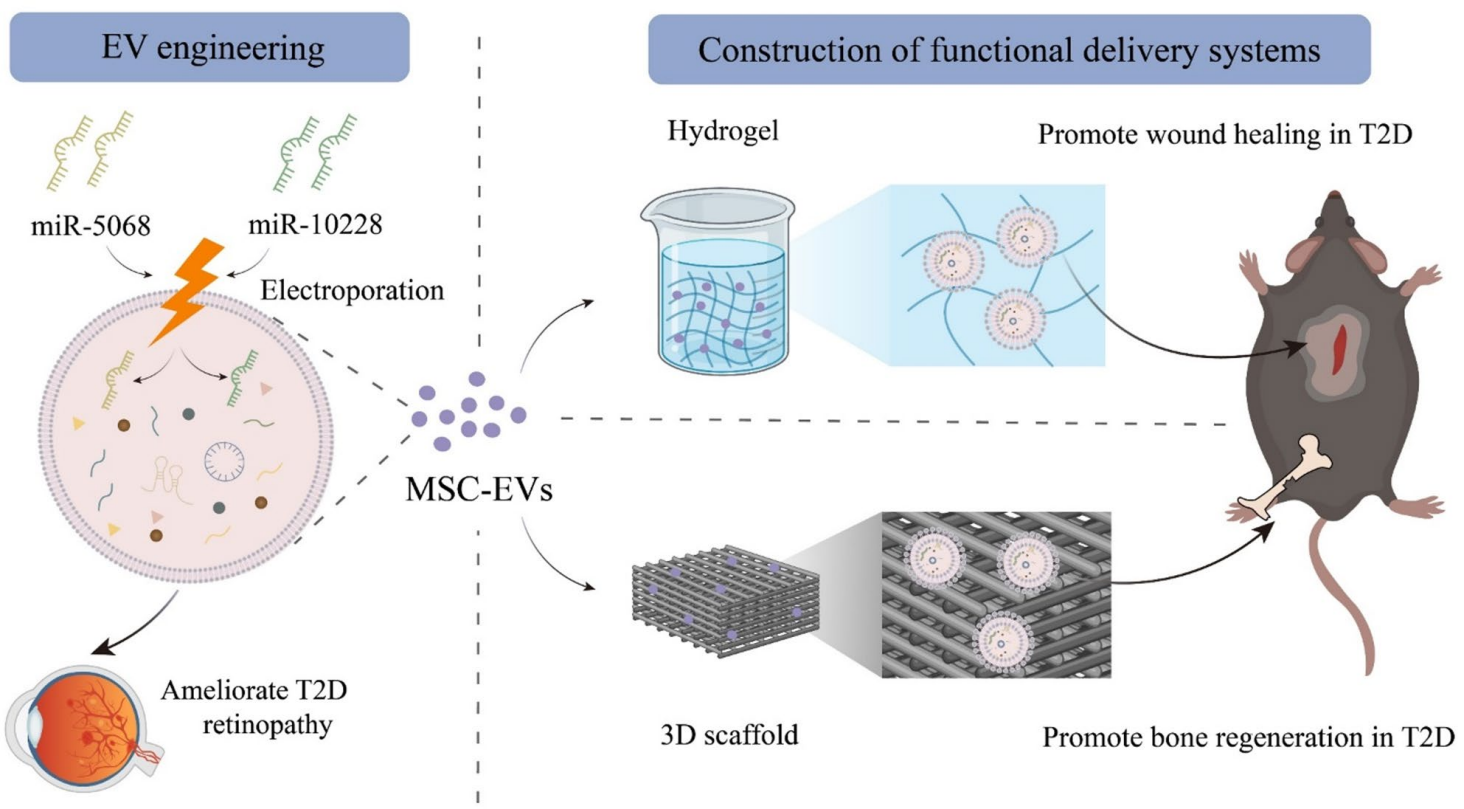
Enhancing the therapeutic efficacy of mesenchymal stem cell-derived extracellular vesicles (MSC-EVs) through engineering strategies. (1) Modifying the nucleic acid content of MSC-EVs *via* electroporation to load specific miRNAs (e.g., miR-5068 and miR-10228), thereby improving their therapeutic effects in conditions like diabetic retinopathy. (2) Constructing functional delivery systems using bioactive materials, such as hydrogels and three-dimensional (3D) scaffolds, to enhance the stability and sustained release of MSC-EVs. These systems improve wound healing and bone regeneration in type 2 diabetes (T2D)

To achieve precise regulation of MSC-EVs for more effective and specific treatments of various diseases, engineering approaches focusing on altering the molecular contents within EVs should be considered (Fig. 8). Studies have found that loading specific microRNAs (e.g., miR-5068 and miR-10228) into MSC-EVs *via* electroporation enhances their therapeutic efficacy to counteract diabetic retinopathy. Experimental data confirm that these engineered MSC-EVs exhibit superior therapeutic effects compared to natural MSC-EVs in improving retinal function, inhibiting retinal cell apoptosis, reducing inflammation, and preventing pathological angiogenesis [11]. Enlightened by this work, MSC-EVs can be customized with therapeutic cargoes based on the patient’s specific disease status and molecular characteristics in the future, which provides a solid foundation for EV-based personalized medicine and precision therapy. Future research should focus on elucidating in-depth the therapeutic targets of EVs, thus further optimizing their applications and accelerating their clinical translation to in treating various diseases.

### Bioactive material-facilitated delivery systems of MSC-EVs

In biomedical engineering, constructing efficient delivery and functional systems of MSC-EVs has become a research hotspot, particularly for improving the EV stability and enhancing their in vivo therapeutic efficacy (Fig. 8). In cutaneous wound healing, innovative carrier designs have brought revolutionary advances to MSC-EV enhancement. For example, incorporating gingival mesenchymal stem cell-derived EVs (GMSC-EVs) into chitosan/silk fibroin hydrogel sponges provides a precise delivery channel for EVs and significantly promotes skin wound healing in T2D. This strategy utilizes and enhances the potential of MSC-EVs in promoting wound repair by stimulating re-epithelialization, collagen deposition, tissue remodeling, vascular and neural growth [99]. Studies have also incorporated UCMSC-EVs into chitosan hydrogels and loaded BMMSC-EVs onto methacrylated gelatin-dopamine hydrogels, which highlight the significant advantages of hydrogel systems in T2D wound therapy. The former ensures the stability of transplanted MSC-EVs and enables the sustained release, maintaining MSC-EV concentration and activity during wound treatment in T2D [100]. The latter further enhances the retention of MSC-EVs at wound sites, significantly improving healing outcomes [101]. These findings suggest that optimizing physical and structural properties of hydrogel carriers can promote the stability and bioactivity of MSC-EVs, providing innovative and effective strategies for treating chronic wounds in T2D. For T2D bone regeneration, 3D-printed gelatin/hyaluronic acid/nano-hydroxyapatite scaffolds combined with MSC-EVs demonstrate a novel method for localized sustainable release of EVs. This method also preserves the stability of transplanted MSC-EVs to significantly increase osteocalcin-positive osteogenic cell numbers in recipients, promoting bone regeneration in T2D [59]. In this design, Gelatin serves as the main component providing scaffold structure and mechanical strength, hyaluronic acid enhances hydration and ECM formation, while nano-hydroxyapatite exhibits excellent bioactivity and osteoconductivity, promoting osteoblast adhesion, proliferation, and differentiation. The scaffold system combining these three components leverages their synergistic advantages, establishing a robust platform for bone tissue engineering and markedly enhancing bone regeneration of MSC-EVs in T2D.

The successful application of sustained release strategies of MSC-EVs in wound healing and bone regeneration indicates broad potential for these approaches in enhancing therapeutic outcomes in T2D, such as neural and cardiovascular complications, meanwhile laying important theoretical and practical foundations for regenerative medicine. Future studies can further explore the combination of different materials and carrier designs to achieve more efficient and sustained release of MSC-EVs, thereby enhancing therapeutic efficacy and reducing potential side effects. Designing carriers of MSC-EVs that target specific diseased tissues may further enable the precise delivery of therapeutic molecules, enhancing efficacy and reducing damages to normal tissues. With ongoing advances in materials science and biomedical engineering, EV delivery systems are expected to play vital roles towards precise, efficient, and personalized medicine in treating T2D and its complications.

## Perspectives: a path to overcome the bottlenecks in MSC-EV clinical translation

MSC-EVs hold great promise for the treatment of T2D, yet their clinical translation lags significantly behind that of MSC-based therapies. This gap primarily stems from three major challenges: (1) Scalable production: the lack of stable and efficient EV preparation systems; (2) Heterogeneity: inherent variability in the structure and function of native EVs, which compromises the predictability of therapeutic efficacy; and (3) Safety assessment: insufficient data on the long-term immunogenicity and in vivo pharmacological profiles of engineered EVs. It should be noted that the lack of data on in vivo biodistribution and target organ homing is a critical challenge shared by both native and engineered EVs, directly impacting the understanding of EV therapeutic mechanisms and the optimization of dosing regimens.

Addressing these bottlenecks requires a stepwise approach. First, the establishment of standardized quality control systems is essential. This involves leveraging omics technologies to identify function-related quality markers, enabling batch-to-batch consistency evaluation. Second, engineering strategies must be pursued in parallel with rigorous preclinical safety assessments, systematically investigating EV biodistribution and immunogenicity to inform future clinical trial design. Future research should integrate high-throughput omics technologies to deeply dissect the specific mechanisms of action of distinct EV subpopulations (particularly exosomes and microvesicles) in T2D complications, thereby providing the mechanistic foundation for more precise engineering strategies and therapeutic applications.

Regarding the application of artificial intelligence (AI), its utility is highly dependent on data accumulation. Building on the mechanistic insights gained from multi-omics analyses, AI can serve as an auxiliary tool to mine omics data for optimizing EV yield and targeting, or to simulate culture conditions to narrow experimental screening. However, the evolution of AI from a supporting tool to an intelligent design platform must be grounded in substantial, standardized datasets on EV function and safety. In the long run, integrating patient multi-omics data may enable AI-driven personalized EV therapies tailored to specific T2D subtypes.

In summary, the clinical translation of MSC-EVs requires a pragmatic path forward—prioritizing standardization, supported by robust preclinical research, and incorporating AI tools with caution—to steadily advance their clinical application.”

## Supplementary Information

Below is the link to the electronic supplementary material.

Supplementary Material 1.

## Data Availability

No datasets were generated or analysed during the current study.
