# Supplementary material for: Mesenchymal stem cell-derived extracellular vesicles in the treatment of type 2 diabetes and its complications: current progress and future directions
Source: Stem Cell Res Ther. 2026 Mar 29;17:178. doi: 10.1186/s13287-026-04991-w (PMC13154470; doi:10.1186/s13287-026-04991-w)
Supplement: Supplementary file 1 — Supplementary Material 1. [file 13287_2026_4991_MOESM1_ESM.docx]

**Supplementary Table 1. List of animal studies examining the therapeutic potential of MSC-EVs on diabetes and its complications.**

| **Species** | **Sex** | **T2D model** | **T2D complication** | **Source of MSC-EVs** | **Autologous/**  **Allogeneic** | **Route of administration** | **Outcome measures** | **Ref.** |
| --- | --- | --- | --- | --- | --- | --- | --- | --- |
| C57BL/6J mice | Male | High-fat diet (HFD) for 4 weeks + intraperitoneal injection of streptozotocin (STZ) (40 mg/kg) | Diabetic wounds | UCMSC | Allogeneic | Local injection into the wound site | Wound healing assessment; Oxidative stress indicators; Vascular function assessment | [63] |
| C57BL/6J mice | Male | High-glucose and HFD for 6 weeks + intraperitoneal injection of STZ (45 mg/kg) | Diabetic foot ulcer | BMMSC | Allogeneic | Local injection into the skin around the wound | Wound healing assessment; Histological analysis; Protein and molecular expression; Non-coding RNA expression; Cell apoptosis analysis; Inflammatory cytokines; Angiogenesis | [70] |
| C57BL/6 mice | Male | HFD (60%) for 12 weeks | Not reported | BMMSC | Allogeneic | Intraperitoneal injection | Body weight and composition; Daily food intake; Glycemic parameters; Insulin and function assessment; Histological analysis; Protein and molecular expression; Serum inflammatory factors | [28] |
| C57BL/6 mice | Male | HFD (60%) for 8 weeks | Not reported | BMMSC | Allogeneic | Intravenous injection | Glycemic parameters; Insulin and functional assessment; Histological analysis; Protein and molecular expression; Inflammatory factors | [81] |
| C57/BL6J mice | Not specified | 18-month-old aged mice | Not reported | BMMSC | Allogeneic | Local intra-bone marrow injection | Glycemic parameters; Insulin and functional assessment; Protein and molecular expression | [90] |
| BALB/C mice | Male | HFD for 6 weeks with intraperitoneal injection of STZ (40 mg/kg) for 5 consecutive days | Diabetic wounds | BMMSC | Allogeneic | Intravenous injection | Wound healing assessment; Histological analysis | [33] |
| BALB/c nude mice | Not specified | HFD (45%) for 5 weeks + intraperitoneal injection of STZ (35 mg/kg) | Diabetic wounds | ADMSC | Allogeneic | Local subcutaneous injection into the wound edge | Wound healing assessment; Histological analysis; Inflammatory factors; Chemokines; Extracellular matrix formation | [87] |
| db/db mice | Male | Genetic model (leptin receptor mutation) | Diabetic wounds | UCMSC | Allogeneic | Injection around the wounds at four sites | Wound healing rate; Histological analysis; Angiogenesis assessment; Blood perfusion | [58] |
| db/db mice | Male | Genetic model (leptin receptor mutation) | Diabetic wounds | UCMSC | Allogeneic | Subcutaneous injection at four points around the wound bed | Wound healing assessment; Histological analysis; Angiogenesis indicators | [91] |
| db/db mice | Male | Genetic model (leptin receptor mutation) | Diabetic wounds | UCMSC | Allogeneic | Local application, using ApoEVs embedded in PF-127 hydrogel applied topically to the wound | Wound healing assessment; Histological analysis; Protein expression; Inflammatory factors; Oxidative stress indicators | [82] |
| db/db mice | Male | Genetic model (leptin receptor mutation) | Diabetic wounds | BMMSC | Allogeneic | Local subcutaneous injection into the wound edge | Wound healing assessment; Histological analysis; Protein expression; Autophagy function evaluation | [88] |
| db/db mice | Male | Genetic model (leptin receptor mutation) | Diabetic wounds | PMSC | Allogeneic | Local injection around the wounds at four injection sites | Wound healing assessment; Histological analysis | [77] |
| db/db mice | Not specified | Genetic model (leptin receptor mutation) | Diabetic wounds | BMMSC | Allogeneic | Peri-wound injection | Wound healing assessment; Histological analysis | [92] |
| db/db mice | Not specified | Genetic model (leptin receptor mutation) | Diabetic wounds | ADMSC | Allogeneic | Topical application onto the wound | In vivo imaging; Wound healing assessment; Histological analysis; Immunofluorescence; Protein expression | [84] |
| db/db mice | Not specified | Genetic model (leptin receptor mutation) | Diabetic wounds | ADMSC | Allogeneic | Subcutaneous injection into the wound | Wound healing assessment; Histological analysis; Oxidative stress indicators; Angiogenesis; Inflammation-related parameters | [31] |
| db/db mice | Male | Genetic model (leptin receptor mutation) | Microangiopathy | UCMSC | Allogeneic | Intravenous injection | Physiological Parameters; Microvascular function and integrity; Angiogenesis assessment; Proteomics and molecular expression; Bioinformatic analysis | [60] |
| db/db mice | Male | Genetic model (leptin receptor mutation) | Diabetic peripheral neuropathy | BMMSC | Allogeneic | Intravenous injection | Neurological outcome; Blood flow perfusion; Intraepidermal nerve fibre density; Axonal myelination; Immunofluorescence image; Protein and molecular expression; Serum inflammatory cytokines | [69] |
| db/db mice | Male | Genetic model (leptin receptor mutation) | Diabetic peripheral neuropathy | BMMSC | Allogeneic | Intravenous injection | Glycemic parameters; Blood lipid indicators; Insulin and function assessment; Neurological function assessment; Neurovascular function; Histological analysis; Protein and molecular expression; Inflammatory factor | [94] |
| db/db mice | Male | Genetic model (leptin receptor mutation) | Diabetic nephropathy | ADMSC | Allogeneic | Intravenous injection | Renal function indicators; Histological analysis; Protein and molecular expression; Cell apoptosis analysis | [73] |
| db/db mice | Male | Genetic model (leptin receptor mutation) | Diabetic limb ischemia | ADMSC | Allogeneic | Intramuscular injection into ischemic hindlimb | Blood perfusion assessment; Histological analysis; Protein expression | [93] |
| db/db mice; SD rat | Male | Genetic model (leptin receptor mutation); Intraperitoneal injection of STZ (35 mg/kg) | Diabetic wounds | BMMSC | Allogeneic | Local multisite subcutaneous injection into the wound | Wound healing assessment; Histological analysis; Protein expression; Angiogenesis and collagen synthesis | [89] |
| db/db mice; SD rat | Not specified | Genetic model (leptin receptor mutation); HFD (45%) for 4 weeks + intravenous injection of STZ (35 mg/kg) | Diabetic retinopathy | UCMSC | Allogeneic | Intravitreal injection | Retinal function assessment; Histological analysis; Protein and molecular expression; Oxidative stress indicators; Vascular function assessment; Cellular functional assays | [11] |
| SD rat | Male | HFD for 8 weeks + intraperitoneal injections of STZ (40 mg/kg) | Diabetic wounds | BMMSC | Allogeneic | Injection around wounds | Wound healing assessment; Histological analysis | [97] |
| SD rat | Male | High sucrose and HFD for 10 weeks + intraperitoneal injection of STZ (35 mg/kg) | Diabetic wounds | GMSC | Allogeneic | Covering the wound with EV-loaded hydrogel | Wound healing assessment; Histological analysis; Nerve fiber density; Microvessel density; Observation of general animal condition | [95] |
| SD rat | Not specified | High-sucrose and HFD for 10 weeks + intraperitoneal injections of STZ (35 mg/kg) | Diabetic foot ulcers | UCMSC | Allogeneic | Covering the wound with EV-loaded hydrogel | Wound healing assessment; Histological analysis | [96] |
| SD rat | Male | HFD (45%) for 4 weeks + intravenous injection of STZ (35 mg/kg) | Diabetic retinopathy | UCMSC | Allogeneic | Intravitreal injection | Retinal function assessment; Histological analysis; Protein and molecular expression; Oxidative stress indicators; Cellular functional assays | [62] |
| SD rat | Male | HFD for one month + intraperitoneal injection of STZ (40 mg/kg) | Bone defect nonunion | BMMSC | Allogeneic | Injection into the bone defect site | Micro-CT analysis; Immunohistochemistry; Histological analysis; Mineralization evaluation | [12] |
| SD rat | Male | HFD for 4 weeks + STZ (30 mg/kg) injection | Calvarial defects | BMMSC | Allogeneic | Scaffold implantation | Micro-CT analysis; Histological analysis | [57] |
| SD rat | Male | HFD for 7 weeks + nicotinamide (120 mg/kg) + intraperitoneal injection of STZ (60 mg/kg) | Not reported | UCMSC | Allogeneic | Intravenous injection | Cellular functional assays; Glycemic parameters; Insulin and functional assessment; Hematological indicators; Blood biochemical indicators of liver and kidney function; Histological analysis | [27] |
| SD rat | Male | HFD (45%) for 5 weeks + intravenous injection of STZ (35 mg/kg) | Not reported | UCMSC | Allogeneic | Intravenous injection | Glycemic parameters; Cellular functional assay; Histological analysis; Protein and molecular expression; islet morphology and function; glucose uptake and metabolism; Serum Inflammatory Cytokines; Safety Indicators | [29] |
| Wistar rats | Male | HFD for 4 weeks + intraperitoneal injection of STZ (35 mg/kg) | Stroke | BMMSC | Allogeneic | Intravenous injection | Neurological function assessment; Histological analysis; Serum miR-9 expression; Inflammatory factors | [83] |
| Gli1-CreER^T2^; mice | Male | HFD for 4 weeks + intraperitoneal injection of STZ (50 mg/kg) | Diabetic wounds | SHED | Allogeneic | Applied with a sterile dressing | Wound healing assessment; Histological analysis | [94] |
